# Comparative Clinical Study of Percutaneous Epididymal Sperm Aspiration and Testicular Biopsy in the Outcome of ICSI-Assisted Fertility Treatment in Patients with Obstructive Azoospermia

**DOI:** 10.3389/fsurg.2022.901601

**Published:** 2022-06-10

**Authors:** Lian Li, Hongqing Liao, Meiqing Li, Jianghua Xiao, Lei Wu

**Affiliations:** ^1^ The Second Affiliated Hospital, Reproductive Center, Hengyang Medical School, University of South China, Hengyang, China; ^2^ Hengyang Nanhua Xinghui Reproductive Health Hospital, Reproductive Center, Hengyang, China; ^3^ Department of Urology Surgery, The Second Affiliated Hospital, Hengyang Medical School, University of South China, Hengyang, China

**Keywords:** obstructive azoospermia, percutaneous epididymal sperm aspiration, testicular sperm aspiration, ICSI for fertility, pregnancy outcome

## Abstract

**Objective:**

To compare and contrast the effects of percutaneous epididymal sperm aspiration (PESA) and testicular sperm aspiration (TESA) on the outcome of intracytoplasmic sperm injection (ICSI)-assisted fertility treatment in patients with obstructive azoospermia.

**Methods:**

Patients with obstructive azoospermia with an age distribution of 20–36 years admitted to the male department of the Reproductive Center of the Second Affiliated Hospital of South China University (Hengyang Nanhua Xing Hui Reproductive Health Hospital) from December 2018 to December 2020 were used in this study. One group was set up as the PESA group to perform PESA, and the other group was set up as the TESA group to perform percutaneous testicular biopsy for sperm extraction. Patients who were unsuccessful in PESA continued to undergo TESA, and if sperm were retrieved, they were classified as the TESA group. General information on male patients and their partners was collected and compared in patients from different sperm source groups. Embryo development (normal fertilization rate, high-quality embryo rate, and high-quality blastocyst rate) and pregnancy outcome (clinical pregnancy rate, miscarriage rate, and ectopic pregnancy rate) were compared between the two groups.

**Results:**

Finally, there were 26 patients in the PESA group and 31 patients in the TESA group. There were no significant differences in terms of age, years of infertility, testosterone level, (FSH) follicle-stimulating hormone level, and testicular volume between the male patients in the PESA and TESA groups of two different sperm sources, and no significant differences were found in the general conditions of the female patients in terms of age, number of eggs obtained, number of sinus follicles, basal FSH value, and basal E2 value (*p* > 0.05). The rate of high-quality blastocysts in the TESA group was significantly higher than that in the PESA group (*p* < 0.05); the differences in clinical normal fertilization rate, high-quality embryo rate, clinical pregnancy rate, miscarriage rate, and ectopic pregnancy rate between the two groups were not statistically significant (*p* > 0.05).

**Conclusion:**

ICSI with different sources of sperm in patients with male factor infertility alone, which had no significant effect on embryo development, embryo implantation rate, clinical pregnancy rate, and miscarriage rate, resulting in better clinical outcomes.

## Introduction

Male infertility is a complex clinical syndrome that accounts for 40%–50% of fertility abnormalities and is the result of multiple diseases or factors, among which azoospermia is one of the common causes of male infertility (1, 2). Men with three consecutive and intermittent sperm retrievals, with not a single sperm found under the microscope after routine semen examination and centrifugal sedimentation, are called azoospermia, which accounts for about 15%–20% of male infertility patients (3). The causes of azoospermia are summarized into two main types: one is spermatogenic dysfunction of the testes themselves such as genetic abnormalities and endocrine disorders resulting in testicular spermatogenic dysfunction, thus preventing sperm production leading to male infertility, called primary azoospermia or non-obstructive azoospermia. The second is obstructive azoospermia, in which the spermatozoa occur normally in the testes of such patients and cannot be excreted normally due to an obstruction of the vas deferens, including the intratesticular, epididymal, vas deferens and ejaculatory duct openings, due to urogenital tract infection, congenital anomalies, or trauma (4, 5).

Intracytoplasmic sperm injection (ICSI), an assisted fertilization technique in which sperm is injected directly into the oocyte plasma, is currently the mainstay of treatment for male infertility (6). This technique fertilizes sperm by injecting them directly into the oocyte plasma of the oocyte through microinsemination techniques, using sperm from the epididymis or testes in addition to naturally ejaculated sperm (7). There are various methods of obtaining sperm from the testes for the diagnosis of azoospermia, such as microsurgical epididymal sperm aspiration (8), percutaneous epididymal sperm aspiration (PESA) (9), testicular biopsy sperm aspiration (TESA) (10), and testicular fine needle aspiration (11) (TFNA), among which PESA and TESA have the advantages of less trauma, lower cost, and better results. The practice of using PESA or TESA to obtain sperm when using self-seminal ICSI for assisted reproduction varies among assisted reproduction facilities, and the fertility outcomes after sperm acquisition for ICSI-assisted fertility treatment reported in the relevant literature (12–14) are also controversial. Therefore, this study provides a comprehensive analysis of the treatment outcomes of PESA- and TESA-obtained sperm applied to patients with obstructive azoospermia undergoing ICSI-assisted fertility, with the aim of further clarifying the priority levels of the two sperm retrieval procedures.

## Materials and Methods

### Subjects

Patients with obstructive azoospermia admitted to the male department of the Reproductive Center of the Second Affiliated Hospital of South China University (Hengyang Nanhua Xing Hui Reproductive Health Specialist Hospital) were included in this study. The diagnostic criteria were as follows: (i) There should be two sperm retrievals, and no sperm should have been found under the microscope after routine semen examination and centrifugal sedimentation; (ii) Bilateral testes should be of normal size and bilateral epididymis should be in normal position; (iii) Sex hormones such as follicle-stimulating hormone (FSH) and Inhibin B (INHB) should be in the normal range; (iv) No abnormality should be seen during peripheral blood karyotype analysis and the azoospermia factor (AZF) gene test; (v) Ultrasound should show grid-like changes in the epididymal duct. The inclusion criteria were also follows: (i) Those who agreed and cooperated with this study; (ii) Those whose spouses had no abnormalities or whose infertility factors were only tubal factors. The exclusion criteria were as follows: (i) Chromosomal abnormalities in either the patient or the spouse; (ii) Acute uterus, endometriosis, or hydrosalpinx in the patient’s spouse; (iii) Poor ovarian response with <3 eggs obtained.

### General Data Collection

Clinical data related to infertility were recorded for each case, including the age of the couple, the number of years of infertility, and the collection of information on the patient’s infertility-related tests. Among them, the definition of infertility was based on the WHO (15)-recommended criteria. According to this definition, male infertility is a result of a couple who have lived together for more than 1 year without using any contraceptive measures and the female partner is infertile due to the male partner.

### Case Grouping

Patients with obstructive azoospermia admitted from the period December 2018 to December 2020 with an age distribution of 20–36 years according to the inclusion criteria were divided into two groups according to the randomized control principle. One group was set up as a PESA group to perform percutaneous epididymal puncture for sperm extraction, and the other group was set up as a TESA group to perform percutaneous testicular biopsy for sperm extraction. Patients who had unsuccessful PESA were continued with TESA, and if sperm was retrieved, they were classified as the TESA group. All enrolled cases were confirmed by temporary sperm retrieval at the time of ICSI on the day of egg retrieval by the female partner and one preoperative sperm retrieval. Finally, there were 26 patients in the PESA group and 31 patients in the TESA group.

### Superovulation Protocol

Different protocols were used for superovulation according to the patients’ own hormone levels and sinus follicles. Human chorionic gonadotropin (HCG) of 5,000–10,000 IU (Manufacturer: Ningbo Renjian Pharmaceutical Group Co., Ltd, Lot No.: 130117, Specification: 1,000 IU) was injected intramuscularly when ≥1 dominant follicle reached a size of 18 mm in diameter or ≥2 dominant follicles reached 16 mm in diameter. The eggs were retrieved by ultrasound-guided transvaginal posterior vault puncture after 34–36 h. After 2–4 h of incubation, the eggs were degranulated using hyaluronidase, and MII eggs were selected for ICSI.

Epididymal puncture for sperm retrieval: During the operation, the towel was routinely disinfected and the surgeon wore sterile gloves, 2% lidocaine was used for local infiltration anesthesia at the puncture site, and after satisfaction, a scalp needle was used to enter vertically from the puncture site. A 10- ml syringe was repeatedly aspirated until a pale yellow fluid was seen and sent to the laboratory, and a sufficient number of PR spermatozoa were visible on microscopic examination to signal the end of the operation.

### Testicular Biopsy for Sperm Extraction

The routine operation and anesthesia were the same as before. The surgeon held the testis with the left hand, and the right hand held the vas separator forceps to enter the skin vertically from the puncture point. Slightly forcefully, the white membrane was punctured, until there was a sense of breakthrough, and the vas separator forceps was opened and the tissue clamped inside the testis, until the varicocele was pulled out and sent to the laboratory. The microscopic examination showed a sufficient number of NP spermatozoa to signal the end of the operation. The obtained sperm was inseminated by an embryologist with ICSI.

### Fertilization, Cleavage, and Embryo Transfer

Three days after egg retrieval, fresh cleavage embryos were selected for transfer into the uterus or fresh blastocysts were transferred after blastocyst culture, depending on the actual situation of the partner. Frozen oocytes or blastocysts would be transferred as needed. Luteal support was routinely performed, and after 3 to 4 weeks, ultrasound was used to check for a successful pregnancy. The pregnancy outcome was then counted at follow-up.

## Observation Indices

### Laboratory Indices

(i) Normal fertilization rate: The ratio of the number of 2PN oocytes cleaved in each group 72 h after insemination to the number of eggs to be inseminated by ICSI in this group. (ii) High-quality embryo rate: The ratio of embryos with 7–9 cells of grade II or higher to 2PN oocytes at 72 h of insemination. (iii) High-quality blastocyst rate: The ratio of blastocysts rated as 3BB or above to 2PN oocyte cleavage embryos source blastocysts.

### Pregnancy Outcome

(i) Clinical pregnancy rate: the ratio of the number of successful pregnancies to the number of embryos transferred. A positive HCG was measured 14 days after transfer and was considered a biochemical pregnancy. A clinical pregnancy was defined as a gestational sac and fetal heartbeat on ultrasound monitoring 35 days after transfer, which included intrauterine and ectopic pregnancies 3–4 weeks after transfer. (ii) Spontaneous abortion rate: the ratio of the number of abortions to the number of pregnancies in each group, and pregnancy loss occurring within 28 weeks of gestation was considered as spontaneous abortion. (iii) Ectopic pregnancy rate: the ratio of ectopic pregnancies to the number of pregnancies in each group. Ectopic pregnancy was defined as an ectopic gestational sac and/or cardiac pulsation visible by ultrasound and confirmed by postoperative pathology.

## Statistical Methods

SPSS 20.0 software was applied for statistical analysis, and continuous variables were tested by an independent sample *t*-test or nonparametric test according to their distribution. The rates were compared by using the *χ*^2^ test, and *p* < 0.05 was considered statistically significant.

## Results

### Comparison of the General Conditions of Male Patients in Two Different Sperm Source Groups

**Figure 1:**
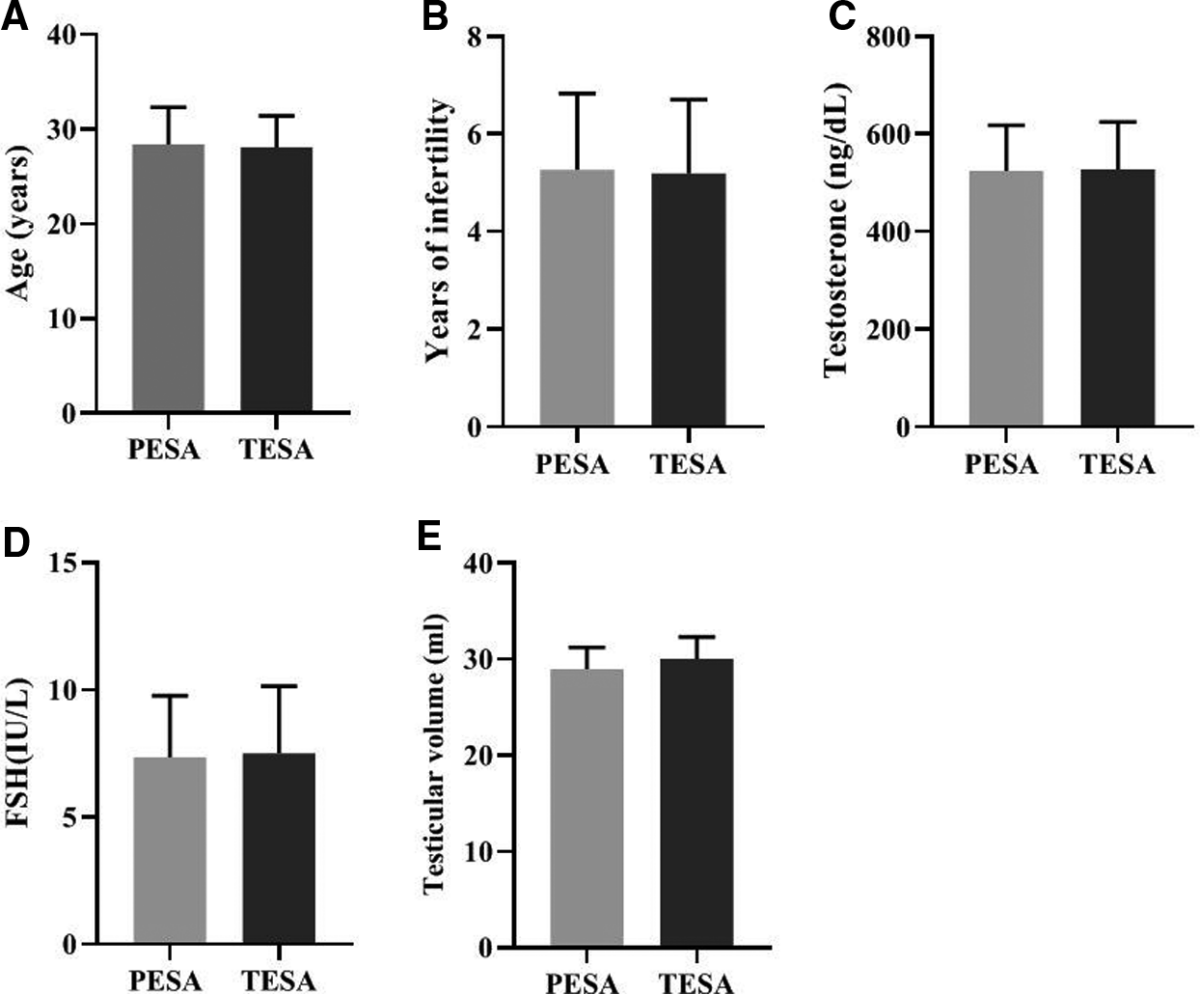
Comparison of the general conditions of male patients in the two different sperm source groups [Mean, Standard deviation (Mean, SD)].

There was no significant difference (*p* > 0.05) in the comparison of the general conditions of male patients in the PESA and TESA groups in terms of age (A), years of infertility (B), testosterone level (C), FSH level (D), and testicular volume (E) between the two different sperm groups (Figure 1).

### Comparison of General Conditions of Female Patients in Two Different Sperm Groups

**Figure 2:**
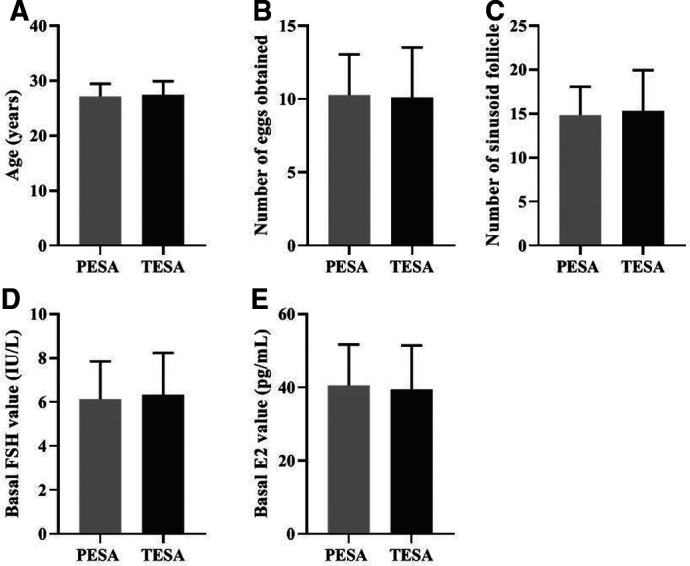
Comparison of the general conditions of female patients in two different sperm groups (Mean, SD).

There were no significant differences in the general conditions of female patients in the PESA and TESA groups between the two different sources of sperm groups in terms of age (A), number of eggs obtained (B), number of sinus follicles (C), basal FSH values (D), and basal E2 values (E) (*p* > 0.05) (Figure 2).

### Comparison of Laboratory Parameters in Patients with Two Different Sources of Sperm Groups

Twenty-six patients underwent PESA and 31 patients underwent TESA, and the frozen embryos were transferred first to blastocysts and then to cleaved embryos. Results: A total of 293 eggs were inseminated by ICSI in the PESA group and 197 2PN fertilized eggs were formed, with a normal fertilization rate of 67.24%; 89 high-quality cleaved embryos, with a high-quality embryo rate of 45.18%; 79 high-quality blastocysts were formed out of 110 blastocysts of 2PN origin with a high-quality blastocyst rate of 71.82%. A total of 378 eggs were fertilized by ICSI in the TESA group, and 278 2PN fertilized eggs were formed, with a normal fertilization rate of 73.54%; 129 high-quality oocytes were formed, with a high-quality embryo rate of 46.40%; 142 high-quality blastocysts were formed among 172 blastocysts of 2PN origin, with a high-quality blastocyst rate of 82.56%. The analysis showed that the rate of high-quality blastocysts in the TESA group was significantly higher than that in the PESA group (*p* < 0.05); the differences in the rates of normal fertilization and high-quality embryos between the two groups were not statistically significant (*p* > 0.05) (Table 1).

**Table 1:** Comparison of the laboratory indices of patients in two different sources of sperm groups (*n*, %).

Information	PESA group	TESA group	*χ*^2^ value	*p-*value
Normal fertilization rate	67.24% (197/293)	73.54% (278/378)	3.178	0.075
High-quality embryo rate	45.18% (89/197)	46.40% (129/278)	0.070	0.792
High-quality blastocyst rate	71.82% (79/110)	82.56% (142/172)	4.565	0.033

### Comparison of Pregnancy Outcomes in Patients with Two Different Sources of Sperm Groups

The PESA group had 18 transferred fresh cleaved embryos, 6 fresh blastocysts, 5 transferred frozen cleaved embryos, and 69 frozen blastocysts, for a total of 98 embryos, with 63 clinical pregnancies (64.29%), 11 spontaneous abortions (17.46%), and 2 ectopic pregnancies (3.17%). There were 14 fresh cleaved embryos and 13 fresh blastocysts, 4 frozen cleaved embryos, and 77 frozen blastocysts, totaling 108 embryos, 63 clinical pregnancies (58.33%), 9 miscarriages (14.29%), and 1 ectopic pregnancy (1.59%). The differences in the clinical pregnancy rate, spontaneous miscarriage rate, and ectopic pregnancy rate between the two groups were not statistically significant (*p* > 0.05) (Table 2).

**Table 2:** Comparison of pregnancy outcomes in patients from two different sperm source groups (*n*, %).

Information	PESA group	TESA group	*χ*^2^ value	*p-*value
Clinical pregnancy rate	64.29% (63/98)	58.33% (63/108)	0.766	0.381
Spontaneous abortion rate	17.46% (11/63)	14.29% (9/63)	0.238	0.626
Ectopic pregnancy rate	3.17% (2/63)	1.59% (1/63)	0.342	0.559

### Observation of Pathological Results

**Figure 3:**
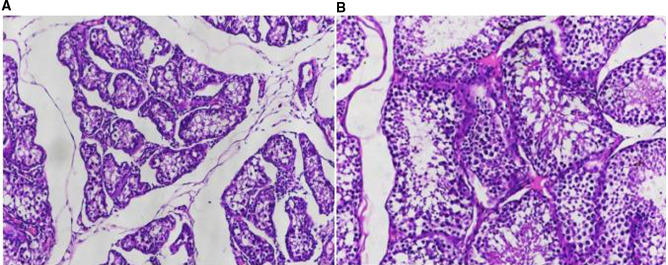
Observation of pathological results. (**A**) HE staining of testicular tissue from a patient with obstructive azoospermia (×20); (**B**) HE staining of testicular tissue from a patient with obstructive azoospermia (×100).

Patient Zheng, who underwent bilateral percutaneous testicular biopsy for sperm extraction in our hospital on 21 April 2019, had bilateral testicular tissue biopsies taken intraoperatively. The microscopic morphology was that spermatogonia at different stages of development were visible in the wall of the seminiferous tubules, and the ratio was basically normal, but the overall number was reduced and the seminiferous epithelium was thinned (Figure 3).

## Discussion

In recent years, the incidence of male infertility has been increasing year after year due to many influences such as the natural and social environment and the general decline of male fertility. The conventional IVF-ET technique offers hope for infertility caused by female factors; however, it has no therapeutic effect on severe male factor and some unexplained infertility (16, 17). The ICSI technique is currently the most effective conception assistance method for the treatment of patients with severe male factor infertility due to its ability to inject a single sperm directly into the mature oocyte and to achieve fertilization, pregnancy, and ultimately their own genetic offspring with only a few sperm (18). Severe oligospermia and weak and malformed spermatozoa are the main indications for ICSI, and with the development of male surgical techniques, patients with obstructive or testicular failure non-obstructive azoospermia can also obtain their own offspring by obtaining sperm in the epididymis or testes for ICSI treatment through percutaneous epididymal sperm aspiration and testicular sperm aspiration (19, 20). The question whether different sperm sources affect the outcome of ICSI treatment has gradually attracted the attention of many scholars, but there is some variation in the findings reported by domestic and international scholars.

Some studies (21) reported no significant differences in the fertilization rate, oogenesis rate, and clinical pregnancy rate between the sperm of different PESA/TESA sources. Some studies (22) also concluded that there was no significant difference in the clinical pregnancy rate between PESA and TESA, while some studies showed that the fertilization rate of sperm from epididymal sources was significantly higher than that of TESA, and the difference in the abortion rate was not significant. The results of Dozortsev et al. (23) concluded that the fertilization rate of PESA from different sperm sources was significantly higher than that of TESA, but the clinical pregnancy rate was significantly lower and the miscarriage rate was higher than that of TESA. High-quality blastocysts are usually able to achieve higher pregnancy and implantation rates, which are important indicators of the developmental potential of transferred embryos *in vivo* (24). With regard to the effect of sperm from different surgical sources on pregnancy outcomes in assisted reproduction, some authors (25, 26) have concluded that there is no significant difference in the effect of testicular and epididymal sperm on clinical pregnancy rates and miscarriage rates. The results of this study showed that the rate of high-quality blastocysts in the TESA group was 82.56%, which was significantly higher than that in the PESA group (71.82%).The higher rate of high-quality blastocysts in the TESA group may be due to the fact that the sperm obtained by the TESA procedure were local fresh sperm from the testicular tissue, while the patients in the PESA group may have had a poor sperm storage microenvironment due to long vasal obstruction, resulting in more sperm DNA fragmentation, which may eventually cause the nucleus of fertilized eggs to develop asynchronously, manifesting as lower-quality blastocysts.

The results of most domestic and international studies (27, 28) showed no statistical difference in the fertilization rate, high-quality embryo rate, miscarriage rate, and ectopic pregnancy rate between TESA and PESA surgical sperm sources. The results of this study also showed no effect of TESA and PESA sperm on the fertilization rate, quality embryo rate, clinical pregnancy rate, early miscarriage rate, and ectopic pregnancy rate in ICSI outcomes. Although early blastocyst quality was slightly worse in the PESA group than in the TESA group, after *in vitro* culture and selection of the highest-quality embryos for transfer, there was no difference in the final pregnancy outcome, and neither epididymal nor testicular sperm had any effect on clinical pregnancy rates. The possible reasons are as follows: the sperm obtained by TESE for ICSI were all morphologically good and motile sperm after laboratory culture treatment, the infertility in both groups was due to purely male factors, both patients had normal fertility and their endometrium had the normal ability to allow embryo implantation, and the quality of oocytes and endocrine conditions were better in the patients’ spouses, who were both under 38 years of age. This shows that there is no significant effect on ICSI pregnancy outcome as long as a certain number of good-quality transferable embryos can be obtained, regardless of whether the patient obtained the sperm from epididymal or testicular puncture (29, 30).

In conclusion, patients with pure male factor infertility can achieve satisfactory clinical pregnancy outcomes either with sperm obtained by the PESA procedure or by applying ICSI with TESA surgical approach sperm. Current studies are mostly limited to the effects of different sperm sources on early embryo quality and pregnancy outcomes after ICSI, and there is a lack of studies on the long-term prognosis of their offspring, still necessitating comprehensive and large samples for long-term follow-up.

## Data Availability

The original contributions presented in the study are included in the article/Supplementary Material; further inquiries can be directed to the corresponding author/s.
